# A Visual Dashboard for Moving Health Technologies From “Lab to Village”

**DOI:** 10.2196/jmir.9.4.e32

**Published:** 2007-10-22

**Authors:** Hassan Masum, Peter A Singer

**Affiliations:** ^1^McLaughlin-Rotman Centre for Global Health, University Health Network, and University of Toronto, MaRS CentreToronto, ONCanada

**Keywords:** Public health, data visualization, Grand Challenges, global health, information dashboard, technology adoption, biotechnology, collaboration, bioethics, lab to village

## Abstract

New technologies are an important way of addressing global health challenges and human development. However, the road for new technologies from “lab to village” is neither simple nor straightforward. Until recently, there has been no conceptual framework for analyzing and addressing the myriad forces and issues involved in moving health technologies from the lab to those who need them. Recently, based on empirical research, we published such a model. In this paper, we focus on extending the model into a dashboard and examine how this dashboard can be used to manage the information related to the path from lab to village. The next step will be for groups interested in global health, and even the public via the Internet, to use the tool to help guide technologies down this tricky path to improve global health and foster human development.

## Introduction

One of the greatest challenges of our time is the inequity in global health. While life expectancies in industrialized countries are 80 years and rising, in many parts of the developing world, particularly Sub-Saharan Africa, they are 40 and falling. The causes and potential solutions to this human tragedy are varied, but science and technology innovation has a role in addressing these inequities.

A flagship initiative dedicated to improving global health using science and technology is the Grand Challenges in Global Health Initiative (GCGH) of the Bill & Melinda Gates Foundation, Foundation for the National Institutes of Health, Wellcome Trust, and Canadian Institutes of Health Research. The GCGH has identified 14 “Grand Challenges” grouped under seven long-term goals: improving childhood vaccines, creating new vaccines, controlling insects that transmit agents of disease, improving nutrition to promote health, improving drug treatment of infectious diseases, curing latent and chronic infection, and measuring health status accurately and economically in developing countries [1]. This initiative has funded 44 research projects to address these challenges, totaling about US $450 million.

**Figure 1:**
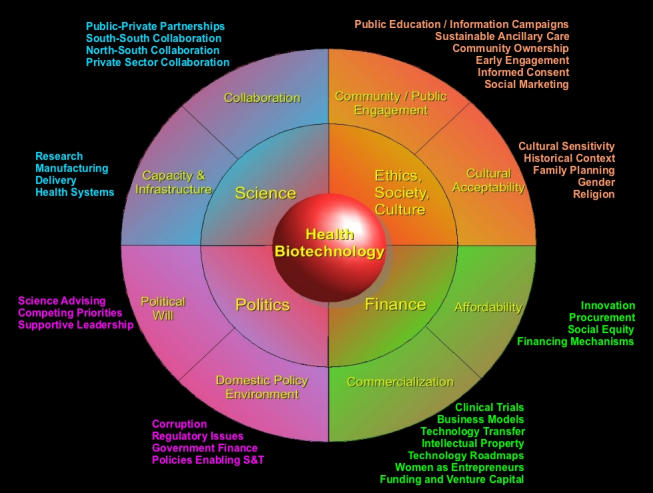
Forces affecting the development and adoption of health biotechnology in developing countries

As these projects, or indeed any research focused on health problems of the poor, move toward developing technologies, serious attention must be paid to how technologies reach those who need them in the developing world—how they move from “lab to village.” To identify the forces that shape the development and adoption of health technologies, we interviewed 70 developing world experts from industry, government, academia, and civil society. The resulting model has recently been published [2] and is shown below in Figure 1. It identifies four major forces—finance, politics, science, and ethics/society/culture—and details many subforces that can facilitate or impede the development and adoption of health technologies in the developing world.

In this paper, we begin to sketch how the model could be applied to guide efforts to move specific technologies from lab to village. In particular, we have developed a series of “dashboard” tools that visually summarize relevant barriers and their status for a given technology area, such as those featured in the GCGH—vaccines, diagnostics, nutritionally enhanced foods, and vector control technologies. This dashboard brings together the various forces affecting adoption success into a single model, which also acts as a summarization of relevant metrics.

A dashboard is defined as a graphical user interface that organizes and presents information in a format that is easy to read and interpret. The effective use of visual models for sharing of qualitative information has been explored both as a general tool [3] and through applications such as in education [4]. Information dashboards in particular have been used in a variety of areas to summarize key metrics for managing complex enterprises so that one can see the most salient information at a glance [5]. The dashboard idea has been used in public health for bioterrorism preparedness [6] and for drug development [7].

The novelty of this paper is that there exists at the moment no tool to analyze and address how to move technologies from lab to village in the developing world. Here we extend our recently published model by turning it into a series of dashboards for this purpose. The work will be useful not only for the GCGH but for anyone interested in the use of information to make decisions about technology adoption, including on the Internet, as we shall illustrate below. 

This paper will explain the evolution and practical use of our dashboard tool and discuss potential developments and applications. We begin by describing our model and then discuss how it can be turned into a dashboard to guide health technology adoption. Next, we show how the dashboard tool can be customized to address specific technologies, differences among geographic regions, and changes over time. Then, we describe how we intend to apply the dashboard tool in our work for the Ethical, Social and Cultural Issues Program of the GCGH [8] in the context of technology-specific working groups, as this simulates how any group interested in technology adoption can use the dashboard model. Finally, consistent with this theme issue’s focus, we discuss future plans for using the Internet to engage a wide array of stakeholders and the public in contributing information on the forces that influence the road from lab to village for health technologies.

## Evolution of a Dashboard

### From a Holistic Model for Health Technology Adoption...

In order to identify the range of issues that influences the development and adoption of health technologies in the developing world, we conducted detailed interviews with 70 key informants from the developing world, the results of which have been recently reported [2] but are reprised below. These 70 interviews yielded a wealth of observations, suggestions, and raw data, covering the gamut from social acceptability of genetically enhanced crops to lessons that systems engineering has for the health field, from investing in local human capital in developing nations to intellectual property regimes around the world.

This raw material provided firsthand insights from the inside on how key players in developing nations see health technologies being developed and adopted (or not). The data were hierarchically categorized into key themes, with the four main categories being finance, politics, science, and ethics/society/culture. Each was, in turn, subdivided, as this partial example illustrates:

Finance

   Affordability

      Innovation

      Procurement

      Social Equity

      Financing Mechanisms

   Commercialization

      Clinical Trials

      Business Models

      ...

Given that any of the four theme areas (finance, politics, science, and ethics/society/culture) could be a significant barrier in a large-scale health implementation effort, a circular diagram intuitively seemed like a reasonable representation to balance the areas. After iterative development, we came up with a detailed multilevel visual taxonomy highlighting key forces affecting development and adoption of health biotechnology (Figure 1).

It is worth emphasizing that this model is empirical in that it was formulated through analysis of a series of one-on-one interviews with several dozen developing world experts. The issues identified are those the experts themselves highlighted as most critical.

The beneficial use of this model as a starting point for discussion is that it provides broadly applicable issues to seed initial discussion. To simply ask a scientist or technology developer “What do you see as some challenges in getting your product adopted?” may not be as effective as systematically going through the model issues in the appropriate level of detail. Informally, we have found that putting this model up on a screen generates immediate interest and discussion.

### ...to a Dashboard...

**Figure 2:**
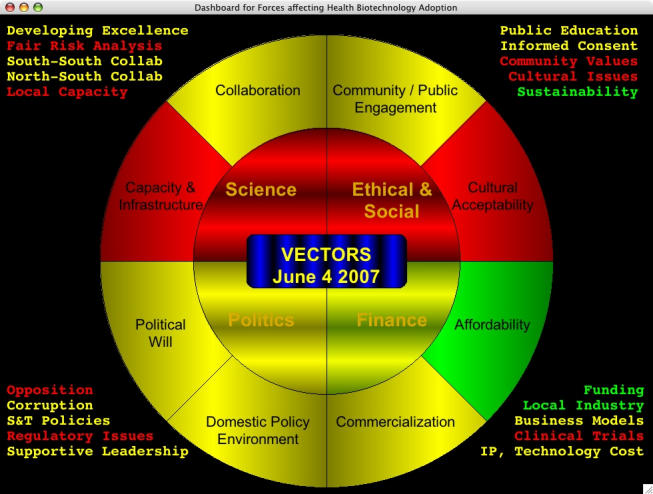
Simulated dashboard for disease vectors

To help illuminate the path for technologies from lab to village, we need to turn the model described above into a dashboard that can be used to represent information on barriers and facilitators of technology adoption. A sample dashboard, using hypothetical data for technologies to control disease vectors, is shown in Figure 2 as an illustrative example.

We found the use of color coding to indicate “threat levels” to be a simple yet effective heuristic—a way of seeing status and trouble spots at a glance. In this illustrative example, each area can be coded with one of three colors: green, yellow, or red. These stand for low risk, medium risk, or high risk, respectively, corresponding to common colors for traffic signals. Here, in one diagram, is an overview of key factors coded by risk level. It is important to note that the colour coding in Figure 2 is based on hypothetical data, essentially the views of our research team. However, the figure illustrates how real data could be used in the dashboard, and below we describe how we intend to do this through the Working Groups in the GCGH. (In the next version of the dashboard, we plan to add a secondary visual motif for the relatively small fraction of the population that is color-blind, just as the position of a light on a traffic signal can tell a color-blind person whether to stop or go.)

The color gradients are not static—they move slowly, generating a subtle background motion that seems to be pleasing to the eye of most viewers. Notice also that there are two distinct gradients: a horizontal one for the quadrants, and a vertical one for the outer octants. As these slowly move, it is made clear without explicit explanation that these are two distinct visual regions and detail levels.

We designed the dashboard by gathering feedback, evaluations, and suggestions in three ways. The first was through iterative development with a closely collaborating team of three people. With regular design reviews and modification requests, the development process was spread over several months in early to mid-2007, on an intermittent basis.

Second, we held several presentation and discussion sessions with the extended Ethical, Social and Cultural Issues team of the GCGH project, several weeks apart. This was done by projecting the model on a large screen in a darkened room, explaining the purpose and application context of the dashboard, and then soliciting oral feedback in a semistructured way; additional participants were located in remote locations in Africa and Asia and took part via audio conference and screen-sharing applications. Question areas included the visual appeal and specific features of the dashboard, the perceived utility in information representation, and the projected workability during Working Group sessions as a tool balancing utility, ease of change, and visual interest to keep in the background for long periods of time. Comments and suggestions were received both orally and through subsequent email communications and one-on-one discussions.

The third method was a presentation and subsequent discussion at a working meeting in Australia in mid-2007, comprising members of the Ethical, Social and Cultural team along with principal investigators and other scientists working on actual GCGH projects. Methodology was similar to the second scenario above but more compressed due to time limitations. Feedback in this case was oral, in the form of open-ended discussion.

The dashboard balances ease of understanding with breadth of factors in a tool that is easily modifiable during a short discussion, yet that contains enough content to be worth publishing as a summary output (along with more detailed recommendations). The dashboard could be thought of as a visual executive summary of barriers and their prioritization.

### ...to Dashboard Sets...

It might be useful to compare different health technologies in terms of their pathways from lab to village. For example, in the GCGH we will create four Working Groups, one each on vaccines, vectors, nutritionally enhanced foods, and diagnostics. Each of the four groups will generate at least one dashboard. Comparing the four may give insight into different pathways of technology adoption and highlight different technology-specific barriers that need to be addressed.

**Figure 3:**
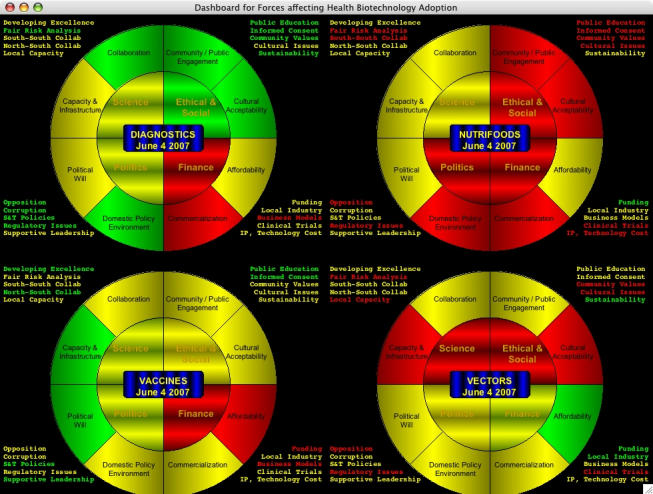
Simulated dashboard set for four health technology areas

In the simulated data set in Figure 3, we see that it is relatively easy to compare the status of the four areas when the dashboards are juxtaposed in this manner. Again, this is hypothetical data based on the views of our research team for illustrative purposes, but it is easy to see how a working group (or even the broader public, as described below) could supply real data to fill out the technology-specific dashboards. The color patterns are simple to grasp and relate to each other, and a single screen can show the status of two dozen barriers for four technology areas.

Since this sort of dashboard set will represent the independent output of four teams in different technology areas, it may be valuable to compare the issues that each team identifies in order to highlight overlaps, commonalities, and differences.

### ...to Cross-Region Comparison...

Cultural issues and concerns of commercialization and capacity differ enormously between Brazil, India, China, and Sub-Saharan Africa. In each area, what issues are most likely to be barriers? Which partners and experts could be brought in? In the course of our previous research looking at innovation at the company level in India [9], and at the country level across several developing nations [10], it has become clear that location-specific knowledge is critical to formulating appropriate strategies and policies.

To address regionalization with dashboards, one could do separate dashboards for each major region. For some issues, general categories of region may suffice (eg, developed, developing, and least-developed nations). For other issues, country-specific analysis may be necessary.

**Figure 4:**
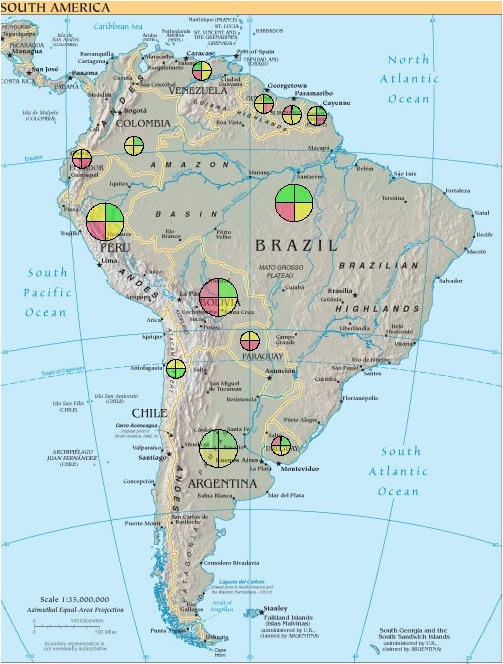
Mockup of cross-regional dashboard for South America

One method we are experimenting with is shrinking the first one or two levels of the dashboard and placing the resulting colorized wheel directly on a geographical map so that one can see major color-coded barrier categories for dozens of countries on a single display. Clicking on a country or region could then show the full dashboard. Figure 4 shows a simulated example for South America. (The idea is that one would start with a world map, drill down through continents, and go down to individual countries or even regions where data are available.)

Two high-graphics examples to learn from are Worldmapper and Gapminder. The Worldmapper project morphs global maps so that the area of each country represents the value of some metric for that country. Health-related examples include money spent on health, working medical staff, infant mortality, HIV/AIDS prevalence, and malaria incidence [11]:

...[W]e are just beginning to learn that an unequal human world is also more likely to be a sick world. How, though, can we better understand the distribution of health resources around the world, and of where most people are sick and die early as compared to people in more privileged positions? How can we fathom the extent to which health equity gaps are growing despite unprecedented global wealth and technological progress? Drawing images is one way to engage more of our imagination to help understand the extent and arrangement of world inequalities in health.

Another model to learn from is Gapminder, by adapting both its flexible country versus indicator layout to summarize and integrate the cross-region comparison qualitative data above and its general design approach of developing a set of tools to make complex statistics around global development issues accessible and even fun to use [12]:

Data in spreadsheets are meaningless to most people. Most statistics are communicated as if musicians stand in front of the audience showing the sheet music instead of playing. We believe the number of users of international development data could multiply by millions if the data was distributed freely on the Internet in interactive and enjoyable graphic interfaces. A much bigger and less skilled audience could thereby understand more complex images of the world.

### ...to Time Series

Opinions shift, technology progresses, and political and financial abilities change. Over several years, dashboards could change as an area evolves. It could then be useful to track versions of a dashboard over time, giving time slices of a multivariate data set. We are exploring adaptations of the dashboard to represent time-varying information.

What about looking forward in time? The International Technology Roadmap for Semiconductors [13] is a technically detailed precompetitive collaboration effort between major players in the semiconductor industry. Among other innovative features, it identifies specific targets that need to be met for continued progress, quantifies them, and expresses them in tables, graphs, and color-coded confidence levels.

For massive health efforts, a similar sort of forward-looking projection could clarify both what we would like to happen and what is currently forecast to happen. The point of identifying barriers is to search for and implement solutions to them, for which timelines, solution dependencies, resource projections, and technology requirements could help keep efforts on track. Along with time, other factors could be included for proposed solutions to barriers, such as cost and human resources.

More generally, causality would be useful to represent—to show, for example, that political will may underlie science capacity. The difficulty is that causality can be complex to analyze; indeed, the direction of causality itself can differ or be a matter of debate between different technology areas. But achieving this holds the possibility of untangling and effectively communicating the “why” behind tough problems.

## Examples of Use

### Working Groups of the GCGH Ethical, Social and Cultural Program

A key motivation for developing these tools was to help Working Groups understand the range and severity of barriers in four Grand Challenge technology areas. Relevant techniques already exist, including technology roadmapping [14], forecasting [15], and foresight and futures methods in general [16]. An application of these techniques to global health is the UK Foresight project on Detection and Identification of Infectious Diseases [17].

But for the Working Groups and similar applications, existing foresight methods need modification to marry technical sophistication with ethical and financial concerns and with creative solutions to downstream barriers, while simultaneously being easy to use and simple to understand. Our aim is to balance these conflicting constraints and develop a generalizable set of tools starting with the dashboard.

Since the use of the dashboard with the Working Groups to date has been targeted not toward a small-screen, PC-based, individual user scenario but toward a large-screen, projector-based, group scenario, visual interest has been a key factor in the design. Our goal has been not to pack the most information possible into a screen but to create a visually appealing representation that stays in the background while remaining interesting enough to periodically refer to—one that will maximize the understanding, clarity, and interest of those who will be referring to the dashboard in the course of group work.

The dashboard will be part of a broader toolkit helping to identify and communicate key barriers for each technology area, including ethical, social, cultural, financial, political, capacity, and collaboration barriers. The end goal of the process is to make it clear what barriers a technology will face from the lab to million-village implementation and what promising solutions to barriers exist, with a clear focus on what will achieve maximal positive health impacts.

As we have seen, the dashboard has been shown and explained to several dozen health and life sciences experts in preparation for use with the Working Groups. The general reaction to date from this informal canvassing has been positive, with people feeling that the dashboard model summarizes a good deal of important information in an easy-to-understand way. At the same time, one reaction was “What comes next? Yes, these are the barriers, and we can see what looks more or less difficult for each area, but what then?”

Use of a convenient tool should not be an excuse for over-simplifying complex issues. Indeed, in-depth discussion and learning requires detailed knowledge, along with the richness of previous successes and failures in the field. One improvement that we plan to make for the Working Group application is to add background information on each item so that clicking on “North-South Collaboration,” for example, would bring up a screen with a short explanation of the barrier and its importance, relevant quotes and references, and links to more information. Additionally, values of this barrier across different health technology areas could be clearly displayed (eg, as a series of aligned bar graphs, forming a complementary cross-area comparison technique to the dashboard set idea shown previously).

Similarly, items could link to a short list of case studies where this issue arose as a problem and suggest best practices for solutions. These would be added as greater understanding of a technology area develops.

### Public Engagement in Health Technology Adoption

Given that the design space of visual tools is complex, we are using feedback from multiple audiences and trial uses for iterative improvement, acknowledging that the tool’s design will benefit from further suggestions by both end users and experts in data visualization. The hypothesis is that providing a single framework containing all the key barriers in health technology adoption will accelerate the understanding and effectiveness of a group tackling a new health technology challenge.

But there is no reason why this process has to be restricted to a small group of co-located experts. A Flash-based version of the dashboard is planned, with online use in mind, so that a larger network of experts and stakeholders could be surveyed for their assessment of technology adoption barriers. The resulting information summary would then be accessible through any Web browser. (This version may entail changes from the current group-oriented version, such as changes to color choices and layout design; what works in a group discussion setting may not be best for an individual user whose goal is to quickly browse through a summary of what is known.)

More generally, the dashboard could be used via the Internet to engage interested citizens in the health technology development process by providing an explanatory platform that could also be used to solicit suggestions. Although there may not be a direct decision-making link between the opinions expressed by citizens and what actually winds up being adopted by technology development organizations, the mere availability of an easy-to-use and informative feedback mechanism may help move citizen opinions upstream in the technology development process.

## Conclusion

At present there is no way to manage the information related to moving new health technologies from the lab to the village. This paper fills that gap by presenting a dashboard for making sense of complex qualitative health technology development data. Tools like the dashboard can help diverse groups talk about barriers systematically and develop a common understanding of which pathways are worth pursuing.

We believe that the dashboard concept could fruitfully be applied to a variety of health planning contexts, especially where issues beyond the purely technical must be considered. For guiding health planning discussions through a range of complex challenges, and communicating these challenges to a broader audience, information design and “maps of the territory” can help chart paths to success for health technologies from lab to village, ensuring that new technologies reach those who need them and thereby addressing, in part, the unconscionable inequities in global health that motivated this global theme issue on poverty and human development.

## Abbreviations

GCGH: Grand Challenges in Global Health Initiative
